# Dynamic increases in p53 expression suppress human myoblast differentiation

**DOI:** 10.3389/fcell.2026.1844660

**Published:** 2026-10-01

**Authors:** Ingrid R. Aragon, Neha Patole, Ajay Vachanaram, Avram Liban, Colin Hogendorn, Ulas Ozkurede, Feng Feng, Darko Bosnakovski, Eric Batchelor, Julia C. Liu

**Affiliations:** 1 Department of Integrative Biology and Physiology, University of Minnesota Medical School – Twin Cities Campus, Minneapolis, MN, United States; 2 Department of Pediatrics, University of Minnesota Medical School – Twin Cities Campus, Minneapolis, MN, United States; 3 Masonic Cancer Center, University of Minnesota Medical School – Twin Cities Campus, Minneapolis, MN, United States

**Keywords:** differentiation, dynamics, myoblast, p53, time-lapse fluorescence microscopy

## Abstract

Skeletal muscle maintenance and regeneration are highly regulated by cell fate decisions made by muscle stem cells and their progeny, myoblasts. Although the transcription factor p53 is well known for its role in cellular stress responses, cell cycle regulation, and tumor prevention, emerging evidence suggests that p53 also plays a critical role in determining whether myoblasts differentiate into muscle fibers or remain single cells that re-enter quiescence to replenish the satellite cell pool. Most previous studies, however, have relied on static, population-level measurements of p53, leaving its dynamic real-time behavior in individual myoblasts largely unexplored. In this study, we use single cell time-lapse microscopy to investigate temporal patterns of p53 activity in fluorescently-tagged immortalized human myoblasts induced to differentiate. We find that cells in which p53 levels increase more quickly and accumulate to higher amplitudes tend to remain single cells rather than differentiate and fuse into myofibers. Understanding how p53 dynamics influence muscle cell fate may provide fundamental insights into muscle maintenance, regeneration, and degenerative disease.

## Introduction

Muscle stem cells, also known as satellite cells, serve as the primary reservoir for skeletal muscle regeneration. These stem cells reside in a specialized niche between the plasma membrane of the muscle fiber and the surrounding basal lamina, where they typically remain in a reversible, non-dividing state known as quiescence (Coller et al., 2006; Mashinchian et al., 2018). Stem cells in quiescence retain the capacity to reenter the cell cycle upon receiving an appropriate stimulus (Tümpel and Rudolph, 2019). In response to muscle injury or activation signals, satellite cells can exit quiescence, reenter the cell cycle, and proliferate to generate myoblasts (Flamini et al., 2018). The potential cell fates of myoblasts include undergoing apoptosis, differentiating into muscle fibers, or returning to quiescence (Cutler and Olwin, 2018; Flamini et al., 2018). Quiescent cells are considered to be in the G0 phase of the cell cycle, which helps protect the stem cell pool by preventing replication-associated DNA damage while maintaining the ability to reenter the cell cycle when needed (Tümpel and Rudolph, 2019). The mechanisms by which muscle satellite cells and their myoblast progeny select from the variety of distinct myoblast fates and the timing at which those decisions are made on an individual cell basis are relatively poorly understood.

Myoblasts express several myogenic regulators that help maintain the balance between differentiation into muscle cells and entry into quiescence as satellite cells. Relatively well-characterized regulators include Pax7, Myf5, and MyoD1. The network of interactions by which these factors interact to commit cells to differentiation and quiescence has been delineated (Motohashi and Asakura, 2012). However, other factors can affect the cell fate decisions that regulate muscle stem cells, and the timing and relative strengths of the impact of these other factors is less understood.

Recently, the transcription factor p53 (Levine, 1997; Vogelstein et al., 2000; Vousden and Lu, 2002; Vousden and Lane, 2007) has been shown to affect the fate of human skeletal myoblasts, determining whether they differentiate into muscle cells, undergo apoptosis, or re-enter quiescence as satellite cells (Yang et al., 2015; Luo et al., 2017; Flamini et al., 2018). A study conducted by Flamini et al. found that elevated p53 levels prevented myoblast differentiation and instead promoted quiescence by inhibiting myogenin, a transcription factor that promotes differentiation of myoblasts into muscle fibers (Flamini et al., 2018). These findings show that p53 regulates myoblast fate and therefore plays an important role in muscle growth and regeneration (Flamini et al., 2018).

The determination that elevated p53 levels affect myoblast differentiation was based on endpoint assays of p53 levels at fixed time point. However, dynamic changes in p53 levels over time have been shown to be dependent on the nature of the p53-activating stimulus and to affect cell fate decisions in several other cellular contexts (Batchelor et al., 2011; Purvis et al., 2012; Chen et al., 2016; Paek et al., 2016; Porter et al., 2017; Reyes et al., 2018; Yang et al., 2018; Harton et al., 2019; Hanson and Batchelor, 2020; 2022; Tsabar et al., 2020; Stewart-Ornstein et al., 2021), including muscle stem cell responses to DNA damage (Yang et al., 2015). For example, in breast carcinoma cells, DNA double strand breaks triggers p53 levels to rise and fall in a series of undamped pulses of relatively constant amplitude and duration regardless of the number of breaks (Lahav et al., 2004; Geva-Zatorsky et al., 2006; Batchelor et al., 2008). In contrast, in response to UV damage p53 levels accumulate in a single pulse that increases to a higher concentration and for a longer duration as the UV dose increases (Batchelor et al., 2011). Distinct cell fate responses were found to be dependent on the form of the dynamics that p53 undergoes in different cell types (Purvis et al., 2012; Paek et al., 2016; Stewart-Ornstein and Lahav, 2017; Yang et al., 2018; Harton et al., 2019; Stewart-Ornstein et al., 2021). These observations suggest p53 dynamics, rather than only static p53 levels, may also be important for controlling muscle cell differentiation, maintenance, and regeneration. Understanding these dynamics is particularly important given that skeletal muscle homeostasis depends on maintaining a proper balance between satellite cell self-renewal and myoblast differentiation (Flamini et al., 2018). Disruptions in this balance contribute to impaired maintenance and regeneration of muscle cells, implicated in conditions such as sarcopenia and muscular dystrophy (Chang et al., 2016).

In this study, using time-lapse fluorescence microscopy we tracked p53 dynamics in myoblasts in response to differentiation signals. We identified distinct p53 responses in cells that differentiated and fused into myofibers and those that remained single cells, with the unfused cells showing a sustained elevated expression of p53 from a relatively early time in the differentiation process. Perturbing p53 expression using a small molecule inhibitor of MDM2, a negative regulator of p53, resulted in elevated p53 levels and subsequent decreases in the percentage of cells differentiating and incorporating into myofibers. Results from this study provide new insights into the mechanisms underlying muscle regeneration and can serve as a foundation for developing therapeutic strategies for muscle degeneration.

## Results

### P53-Venus dynamics are distinct between myoblasts that differentiate and fused into myofibers compared with cells that remain unfused

To track p53 levels in individual myoblasts during the process of differentiation, we expressed a translational fusion of the yellow fluorescent protein Venus to the C-terminus of p53 in immortalized human myoblasts (Geva-Zatorsky et al., 2006; Batchelor et al., 2008). This live cell reporter of p53 levels has been well characterized in numerous studies as an effective system for tracking p53 expression dynamics in single living cells of a variety of cell types (Lahav et al., 2004; Geva-Zatorsky et al., 2006; Batchelor et al., 2008; Loewer et al., 2010; Batchelor et al., 2011; Purvis et al., 2012; Chen et al., 2016; Paek et al., 2016; Porter et al., 2017; Stewart-Ornstein and Lahav, 2017; Stewart-Ornstein and Lahav, 2017; Reyes et al., 2018; Harton et al., 2019; Tsabar et al., 2020; Stewart-Ornstein et al., 2021). The fluorescent reporter has two characteristics particularly noteworthy: 1.) the p53 fluorescent reporter has been shown to directly track levels of the endogenous, wild-type p53 (Lahav et al., 2004; Geva-Zatorsky et al., 2006; Batchelor et al., 2008), and 2.) the fluorescent reporter retains p53’s transcriptional activity to affect downstream cellular responses (Lahav et al., 2004; Loewer et al., 2010; Chen et al., 2016; Paek et al., 2016; Reyes et al., 2018; Harton et al., 2019; Tsabar et al., 2020). To establish the system in myoblasts, we used lentiviral transduction to introduce a previously developed p53-Venus cDNA (Batchelor et al., 2008) into the immortalized clonal human myoblast line M007 (Bosnakovski et al., 2023). We generated clonal M007 cells positive for p53-Venus expression (M007 p53-Venus cells) through antibiotic selection and screening for p53-Venus expression via flow cytometry.

To determine the pattern of p53 dynamics during the process of muscle stem cell differentiation, we tracked p53-Venus levels in individual M007 p53-Venus cells through time-lapse fluorescence microscopy. At the start of image acquisition, the cell medium was switched to medium containing greatly reduced growth factors (“differentiation medium”) to stimulate myoblast differentiation. Images were acquired every 20 min for a total of 3 days, giving sufficient temporal resolution to capture possible p53 dynamic changes (Geva-Zatorsky et al., 2006; Batchelor et al., 2011) over a total length of time during which myoblast differentiation can occur (Bosnakovski et al., 2023). From analysis of acquired bright field images, we could observe two major cell phenotypes: cells that differentiated, fused, and formed myofibers by approximately 2 days following the switch to differentiation medium, and cells that remained unfused throughout the 3 days following introduction of differentiation medium (Supplementary Movies S1, S2).

From the acquired fluorescence images, we tracked and quantified p53-Venus expression in individual cells. P53-Venus expression was observed to increase typically starting between 12 and 24 h. In one subset of cells, those that differentiated and fused into multi-nucleated myofibers, we found that p53-Venus expression remained relatively low throughout the image acquisition (Figure 1A), with either a very small pulse or no detectable increase in p53-Venus expression. In a second subset of cells, however, p53 levels continued to rise to relatively high levels and remain elevated, leading to a monotonic increase or a large single modal pulse over the 3 days of imaging (Figure 1B). This dynamic behavior is qualitatively similar to what has been previously observed for UV-induced p53 responses in transformed cells (Batchelor et al., 2011), and contrasts with the undamped, oscillatory p53 pulses that have been observed in response to DNA double strand breaks in several different cell types (Stewart-Ornstein and Lahav, 2017). These cells were those that remained as individual cells throughout the 3 days of imaging.

**FIGURE 1:**
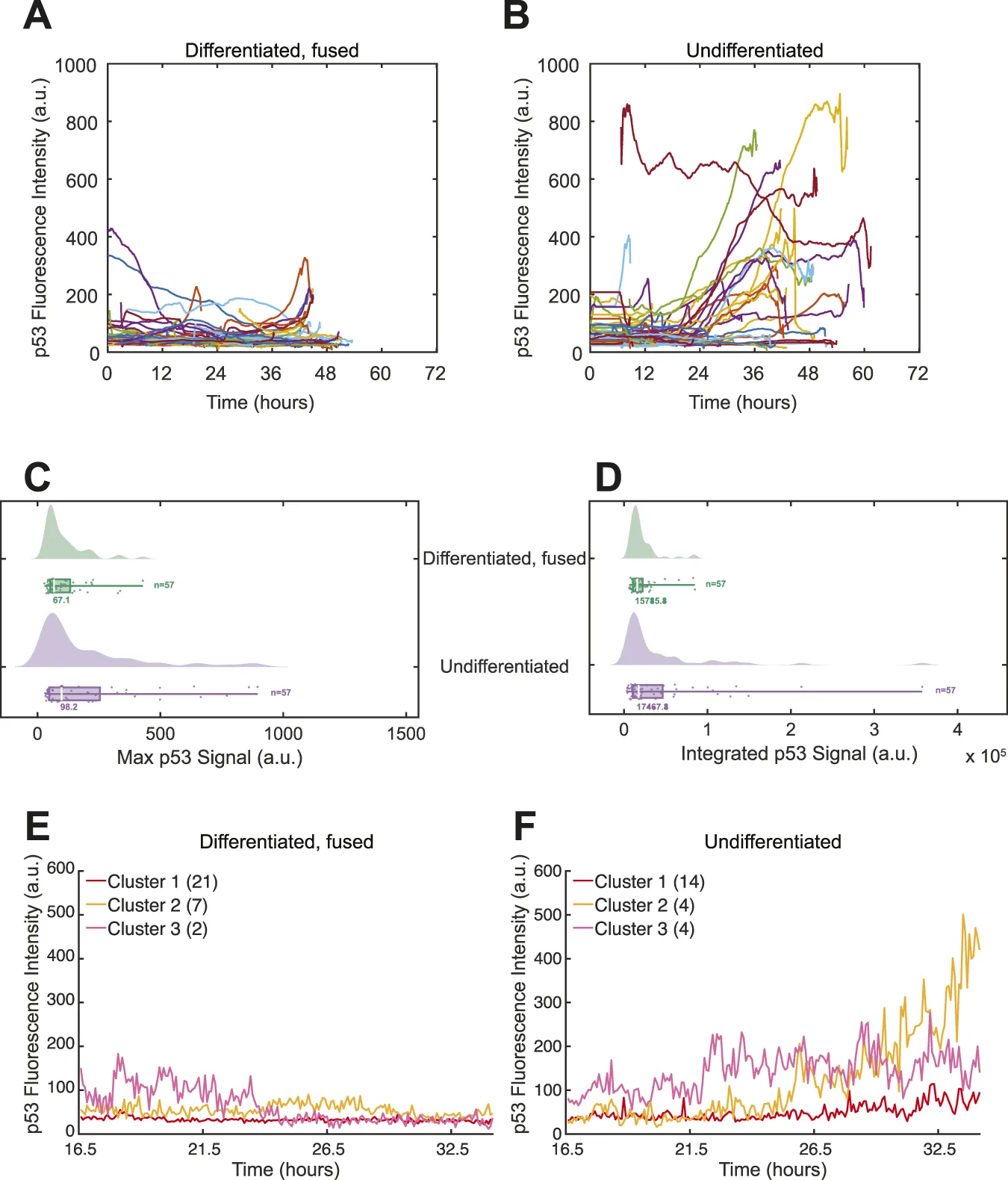
P53 levels in single myoblasts undergo distinct dynamics in cells that remain unfused compared with cells that differentiate into myofibers. **(A,B)** Time-lapse fluorescence microscopy traces of mean nuclear p53-Venus levels in M007 p53-Venus cells treated with differentiation medium for cells that differentiated and fused into myofibers (A, 57 cells) or cells that remained unfused over 72 h (B, 57 cells). Colors are randomly assigned to each trace to visually aid in distinguishing fluorescence traces from individual, distinct cells. Results are from biological triplicates. **(C)** Raincloud plots of maximum p53-Venus levels attained during imaging in cells that remained unfused or that fused. **(D)** Raincloud plots of integrated p53-Venus over the time of imaging for cells that remained unfused or that fused. **(E–F)** Clustering analysis of M007 p53-Venus cells that differentiated and fused into myofibers. **(E)** or remained unfused (F, clustering over time points 0 – 33.5 h) following treatment with differentiation medium. Number of cells in each cluster are indicated in each legend.

Qualitative inspection found that the distinct p53 dynamic behaviors were separately associated with distinct cell fates. The cells that had relatively higher levels of p53 expression tended to remain as isolated individual cells. In contrast, cells with relatively low accumulation of p53 or no detectable changes in p53 expression tended to differentiate and fuse into myofibers (Supplementary Movie S1). Quantification of maximum p53 values found a median of 98.2 a. u. (arbitrary units of fluorescence–a. u.) for cells that remained isolated throughout the imaging compared with 67.1 a. u. for cells that fused into fibers (Figure 1C). Statistical comparison of the two distributions resulted in a p-value of 0.052, suggesting a trend that the dynamic feature of maximum p53 level can distinguish between the two cell fates, albeit without achieving statistical significance. However, integrated levels of p53 were significantly higher in unfused cells (17,468 a. u.) compared with fused cells (15,786 a. u., p-value 0.03 by two-sample Kolmogorov-Smirnov test) (Figure 1D), which is consistent with the integrated values of p53 expression being a driving feature of cell fate decisions in distinct cellular contexts (Purvis et al., 2012; Paek et al., 2016).

### Clustering of p53-Venus dynamics suggests commitment to an unfused myoblast state occurs with relatively early upregulation of p53 expression

To identify other potential differences between the p53 dynamics of unfused myoblasts compared with fused myoblasts, we performed unsupervised clustering of the traces (Figures 1E,F). To better identify potential differences in dynamic upregulation of p53, we specifically clustered the dynamic traces on time points over which p53 dynamics began to increase over baseline levels following the introduction of differentiation medium (16.5 – 33.5 h). We found that the dynamic traces generated a cluster of cells with a delayed and low level of p53 (cluster 1), as well as two different clusters for highly p53-responsive cells (clusters two and 3) that remained as single cells during the differentiation process (Figure 1F). Qualitatively, both clusters of highly p53-responsive cells had relatively smooth, non-oscillatory pulses, consistent with visual inspection of the full set of traces for each condition. Initial p53 increases in the unfused cell clusters occurred in the range of 16.5 – 28 h, a time preceding the formation of myofibers observed via microscopy (Supplementary Movies S1, S2), supporting a potential role of upregulation of p53 in retaining cells in an unfused state.

### Elevating total p53 levels during differentiation reduces myoblast differentiation and fusion

Given that lower integrated p53 levels correlated with myoblast fusion in response to differentiation conditions (Figure 1D), we sought to determine if pharmacologically elevating p53 levels would reduce the levels of myoblast fusion and fiber formation. Concurrent with switching the growth medium of M007-p53Venus cells to differentiation medium, we treated the cells with 15 µM Nutlin-3. This small molecule binds to the E3 ubiquitin ligase MDM2 at the interface of MDM2 with p53, preventing the ubiquitination and subsequent degradation of p53 (Vassilev, 2004). Time-lapse fluorescence microscopy revealed elevated levels of p53 expression over the full acquisition time for both fused and unfused cells (Supplementary Movies S3, S4, Figures 2A,B). Qualitatively, we observed a larger number of cells remain unfused following exposure to Nutlin-3 than in conditions of differentiation without Nutlin-3 (Supplementary Movies S1, S2). Both the peak levels of p53-Venus (Figure 2C) and integrated levels of p53-Venus (Figure 2D) were significantly higher in cells that remained unfused following Nutlin-3 treatment compared with cells that became fused (p-values 0.0001 and <0.0001 b y Kolmogorov-Smirnov two-sample test, respectively).

**FIGURE 2:**
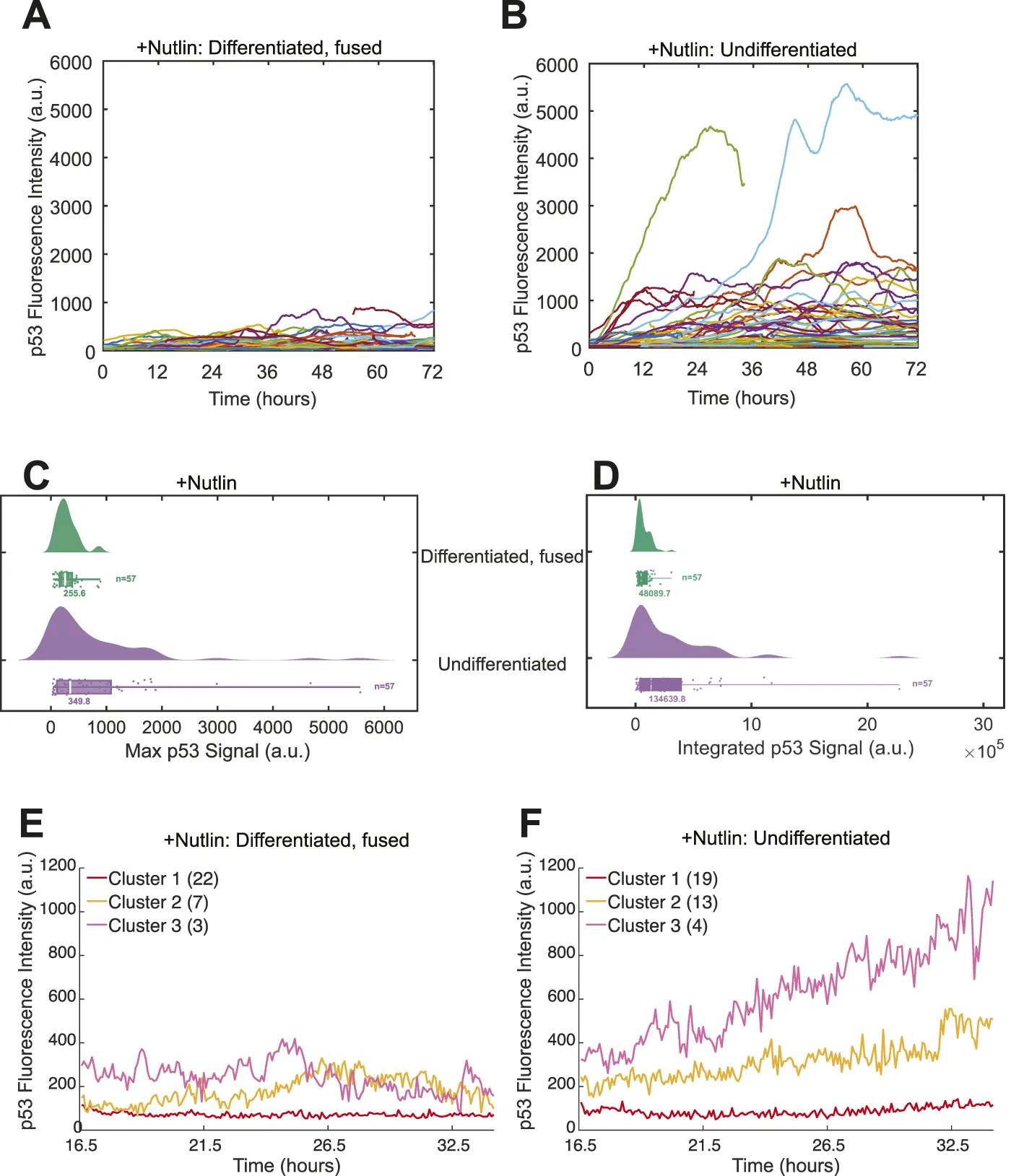
Treatment of myoblasts with Nutlin-3 elevates p53 levels and changes the profile of p53 dynamics in unfused myoblasts. **(A,B)** Time-lapse fluorescence microscopy traces of mean nuclear p53-Venus levels in M007 p53-Venus cells treated with differentiation medium and with 15 µM Nutlin-3 for cells that differentiated and fused into myofibers over 72 h (A, 57 cells) or cells that remained unfused (B, 57 cells). Colors are randomly assigned to each trace to visually aid in distinguishing fluorescence traces from individual, distinct cells. Results are from biological triplicates. **(C)** Raincloud plots of maximum p53-Venus levels attained during imaging in cells that remained unfused or that fused. **(D)** Raincloud plots of integrated p53-Venus over the time of imaging for cells that remained unfused or that fused. **(E,F)** Clustering analysis of M007 p53-Venus cells that differentiated and fused into myofibers **(E)** or remained unfused (F, clustering over time points 0 – 33.5 h) following treatment with differentiation medium and 15 µM Nutlin-3. Number of cells in each cluster are indicated in each legend.

Treatment with Nutlin-3 has been shown to sustain p53 at higher levels, and indeed, in these conditions we observe the emergence of clusters of p53 dynamics with accumulating p53 levels that are elevated relative to the levels in cells without Nutlin-3 exposure for both cells that differentiated and fused into myofibers (Figure 2E compared with Figure 1E) and cells that remained unfused (Figure 2F compared with Figure 1F). For Nutlin-3 treated cells that differentiated and fused, the majority of cells once again maintained relatively low p53 expression (Figure 2E). The two clusters of fused cells with low but elevated p53 expression did not exhibit a monotonic increase. In contrast, cells that remained unfused separated into clusters that had much higher accumulation of p53 with dynamics that were monotonically increasing, qualitatively consistent with p53 dynamics in unfused cells that were not exposed to Nutlin-3 (Figure 1F). Taken together, the clustering analysis provides support that the main dynamic modes of p53 expression in unfused cells are monotonically increasing, sustained elevated expression, or a relatively high amplitude and duration pulse, with or without overall elevation of p53 levels by Nutlin-3 treatment during exposure to differentiation conditions.

We also performed immunofluorescence-based analysis on single cells fixed at the conclusion of live cell imaging to verify that, in support of the microscopy data, endogenous p53 levels correlate with cell fate decisions. One, two, or 3 days post induction of differentiation we fixed cells, stained for total p53 and myosin heavy chain (MYHC, a differentiated muscle cell marker), and quantified protein expression using immunofluorescence microscopy (Figures 3A,B; Supplementary Figures S1A-D). By 3 days, we observed statistically significant elevation of p53 in cells that were unfused and with low MYHC signal (undifferentiated) compared with cells that were visibly fused and with high MYHC levels, in conditions without and with Nutlin-3 treatment (Figures 3C,D, P-values of 0.0326 and 0.0122, respectively, by Kolmogorov two-sample test). We also observed a small population of cells for each condition which remained unfused at the 3-day timepoint but that expressed relatively high levels of the muscle marker MYHC. In this small population of cells, we quantified p53 levels to be intermediate between the unfused/low MYHC cells and the fused/high MYHC cells. Quantification of cell numbers for each of the three categories of cells (Figure 3E) showed that elevation of p53 by Nutlin-3 resulted in an increase in the number of cells remaining unfused, either with low (undifferentiated) or high (differentiated) levels of MYHC, and reduction in myoblast fusion.

**FIGURE 3:**
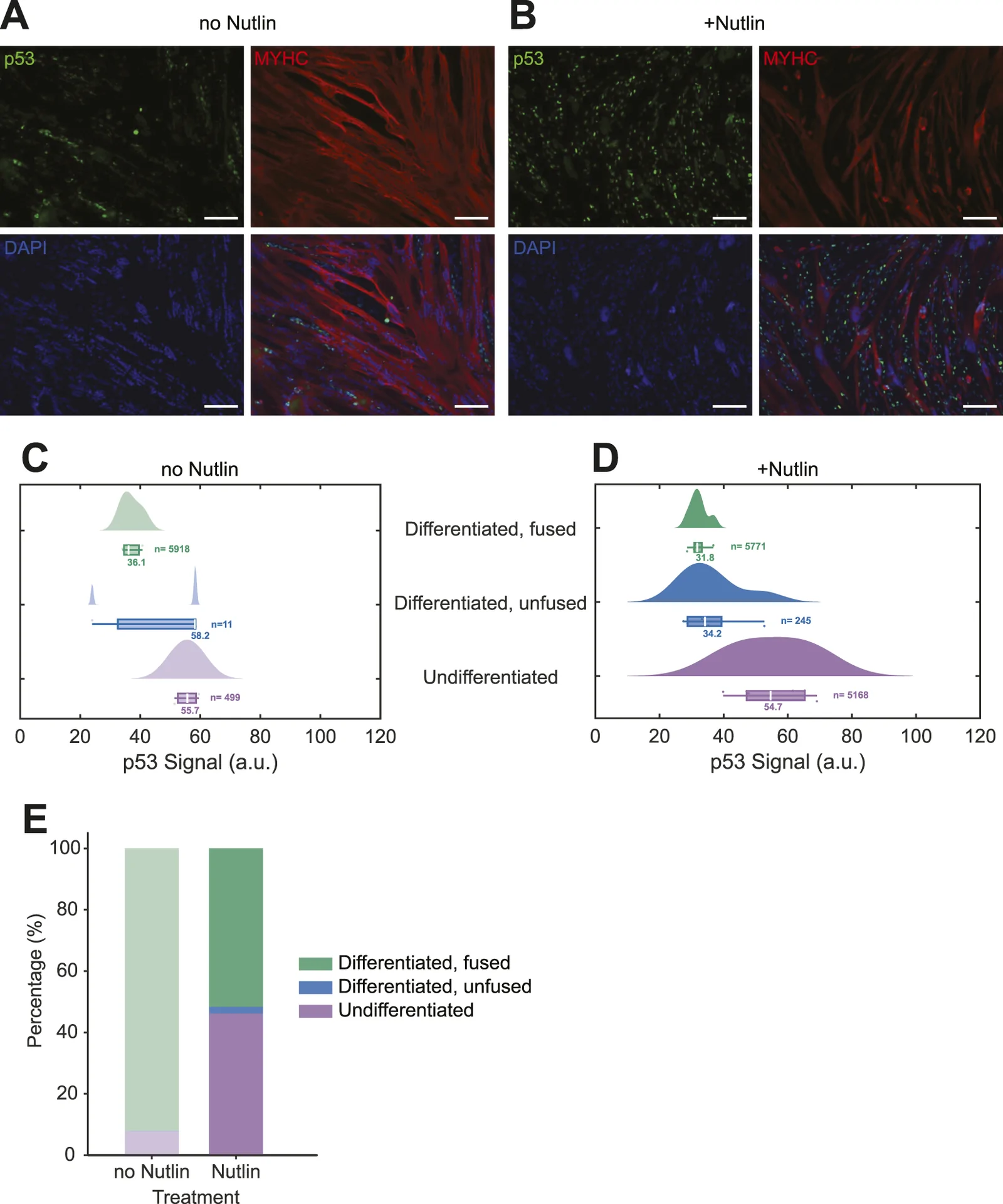
Elevating p53 levels in myoblast differentiation attenuates fusion into myofibers. **(A,B)** Immunofluorescence images of M007 p53-Venus cells stained for the muscle marker MYHC (red), p53 (green), and DAPI (blue) for cells treated with only differentiation medium **(A)** or differentiation medium and 15 µM Nutlin-3 **(B)** for 72 h. Scale bar = 50 µm **(C,D)** Rain cloud plots of p53 levels in individual M007 p53-Venus cells treated with only differentiation medium **(C)** or differentiation medium and 15 µM Nutlin-3 **(D)** for 72 h for cells that were unfused with low MYHC, unfused with high MYHC, or fused with high MYHC. Number of cells analyzed for each subset of cells are indicated to the right of each rain cloud distribution. Results are from biological triplicates. **(E)** Bar plots of the relative cell numbers for cells that were unfused with low MYHC, unfused with high MYHC, or fused with high MYHC.

To investigate how the elevation of p53 in a subset of cells affected cell cycle, we differentiated the M007 p53-Venus cells for 3 days and then used fluorescence-activated cell sorting (FACS) to separate individual cells that expressed elevated levels of p53-Venus (“p53-high” cells consisting of top ∼30% of the sorted population; Supplementary Figure S2) from cells with low p53-Venus expression (“p53-low” cells consisting of the remaining ∼70% of sorted cells; Supplementary Figure S2). Assessing the cell cycle status of these cells by staining with propidium iodide revealed a significant enrichment in a broad G2/M peak and a reduction in the G1 peak of p53-high cells relative to p53-low cells (Figures 4A,B).

**FIGURE 4:**
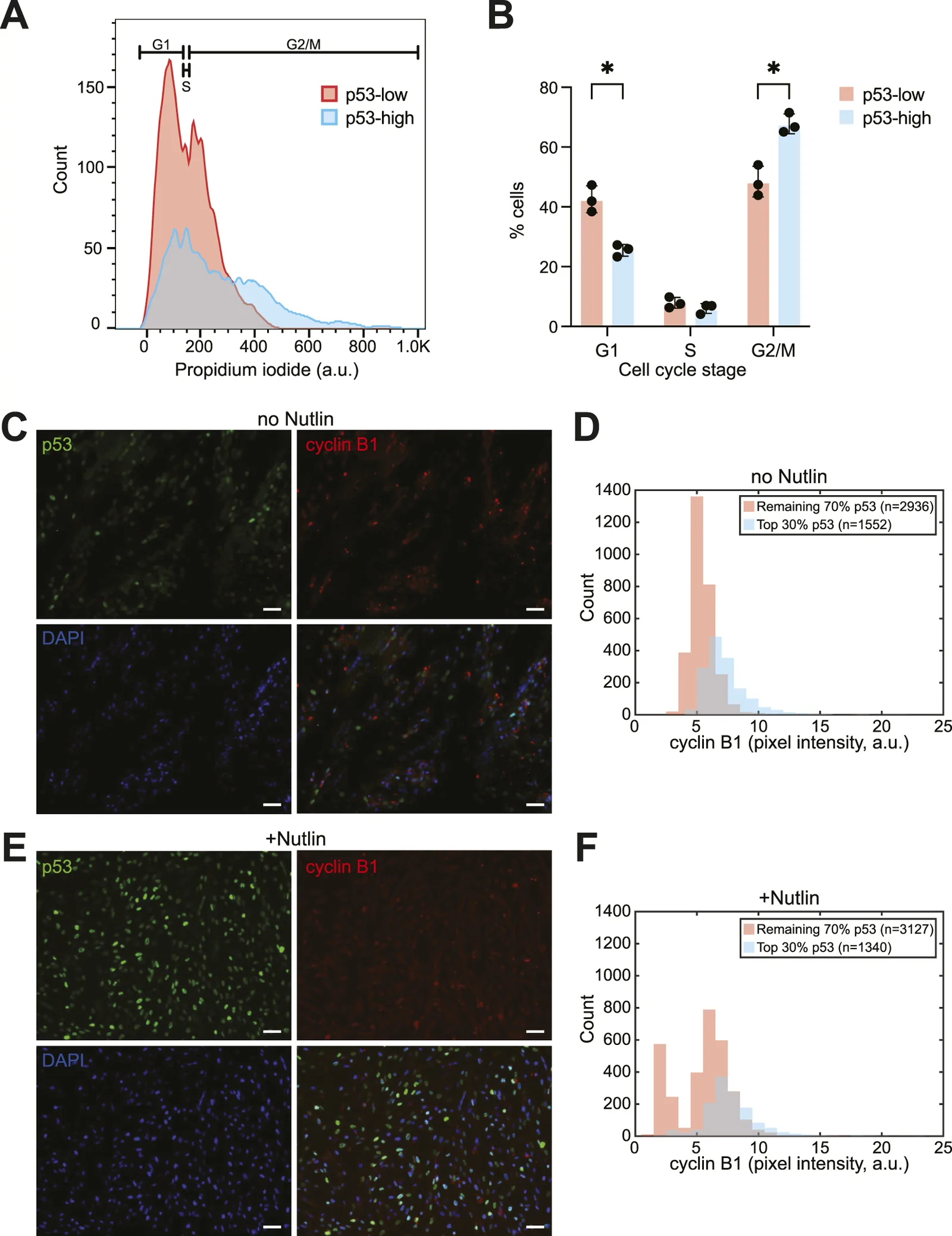
High p53 levels are associated with accumulation of cells in G2/M. **(A)** Propidium iodide staining for DNA content in M007 p53-Venus cells grown in differentiation medium for 72 h and sorted by FACS into populations of cells expressing low (blue) or high (red) p53-Venus expression. Histograms are for one representative biological replicate of the experiment performed in triplicate. **(B)** Quantification of cell cycle stage distributions for the cells in **(A)**. Bars represent mean values for biological triplicates. Error bars are standard errors of the means. * indicates statistical significance with p < 0.01 as determined by multiple unpaired t-tests. **(C)** Representative immunofluorescence images of M007 p53-Venus cells grown for 72 h in differentiation medium and stained for p53 (green), cyclin B (red), and DAPI (blue). Scale bar = 50 µm. **(D)** Quantification of cyclin B levels from immunofluorescence images as in **(C)** for the 30% of cells with the highest p53 expression (1,552 cells) and the remaining cells with the lowest 70% p53 expression (2,936 cells). Results are for 4,488 total cells quantified from biological triplicates; distributions are significantly distinct by Kolmogorov-Smirnov test with a p-value <10^−136^. **(E)** Representative immunofluorescence images of M007 p53-Venus cells grown for 72 h in differentiation medium supplemented with 15 µM Nutlin-3 and stained for p53 (green), cyclin B (red), and DAPI (blue). Scale bar = 50 µm. **(F)** Quantification of cyclin B levels from immunofluorescence images as in **(D)** for the 30% of cells with the highest p53 expression (1,340 cells) and the remaining cells with the lowest 70% p53 expression (3,127 cells). Results are for 4,467 total cells quantified from biological triplicates; distributions are significantly distinct by Kolmogorov-Smirnov test with a p-value <10^−151^.

Based on the FACS analysis, we speculated that the molecular mechanism driving this accumulation of p53-high cells in G2 or M was a p53-mediated cell cycle arrest in G2. Based on previous studies, we identified two potential p53-dependent mechanisms for the G2 arrest: repression of cyclin B1 expression (Innocente et al., 1999) or nuclear sequestration and inactivation of cyclin B1 without the degradation associated with mitotic exit (Krenning et al., 2014). To test if one of these mechanisms is responsible for the accumulation of p53-high cells in G2, we quantified cyclin B1 levels through immunofluorescence staining. If p53-dependent repression of cyclin B1 is responsible for the arrest, we would expect a reduction in cyclin B1 levels in p53-high cells relative to p53-low cells. Alternatively, if p53-dependent nuclear accumulation of cyclin B1 is responsible, we would expect p53-high cells to have elevated levels of nuclear cyclin B1 relative to p53-low cells. We differentiated M007 p53-Venus cells for 3 days, fixed and permeabilized cells, and then performed immunofluorescence staining for both p53 and cyclin B1. Based on our FACS results (Figures 4A,B; Supplementary Figures S2A-B), we focused on the 30% of cells expressing the highest p53 levels, and we found that they were significantly enhanced for nuclear cyclin B1 expression compared with the remaining 70% of cells (Figures 4C,D). This result aligns with p53-dependent nuclear sequestration of cyclin B1 leading to G2/M arrest of p53-high cells, as observed by FACS. We further tested this mechanism by stabilizing p53 expression with Nutlin-3 during differentiation, elevating p53 expression across the cell population (Figure 4E compared with Figure 4C, significant increase of mean nuclear p53 levels from 8.55 to 65.6 AU, p-value <10^−150^ by Kolmogorov Smirnov test). Quantification of nuclear cyclin B1 expression by immunofluorescence staining in the 30% of Nutlin-treated cells with highest p53 expression compared with the remaining 70% of cells again showed a significant increase in nuclear cyclin B1 expression (Figure 4F). These data suggest that p53-dependent nuclear sequestration of cyclin B1 likely contributes to the mechanism by which a subpopulation of cells with elevated p53 resist differentiation cues and remain single cells.

Taken together, our results support a model in which elevated levels of p53 during the process of muscle myoblast differentiation lead to cell cycle arrest and prevent the fusion of cells into mature myofibers, instead maintaining myoblasts as single cells.

## Discussion

The process of muscle repair after injury comprises multiple transitions in cell state, including the re-entry of quiescent satellite cells into the cell cycle to become proliferating myoblasts, and the cell fate decision of myoblasts to differentiate and fuse into myofibers or remain single “reserve” cells that can re-enter quiescence to replenish the satellite cell pool (Abou-Khalil et al., 2013). Simulating the latter decision *in vitro*, we induced differentiation via serum starvation in immortalized human myoblasts while monitoring p53 levels by time-lapse microscopy. As might be expected given the role of p53 dynamics in many other decisions in many cell types, the changes in p53 over time contributed toward the differentiation decision. Rather than oscillatory or pulsatile dynamics, we observed that little to no induction of p53 was associated with cells undergoing fusion to form myofibers, whereas an early or high amplitude pulse of p53 was characteristic of cells remaining unfused. These large, single modal p53 responses bear qualitative similarities to those observed in other cell systems: UV damage of breast carcinoma cells (Batchelor et al., 2011); treatment of cancer cells by continuous exposure to chemotherapeutics (Yang et al., 2018); and artificially maintaining p53 expression through small molecule treatment with Nutlin-3 following DNA double strand break induction (Purvis et al., 2012). In all those studies, monotonic and single modal p53 pulses were found to either correlate with or generate terminal cell fates–apoptosis for UV and chemotherapeutic-induced damage and senescence for Nutlin-3. In contrast, it could be argued that in the myoblast context that low p53 is associated with a post-mitotic cell fate as myotubes mature into muscle fibers, and instead the cells that experienced a large p53 pulse are in a transient state of cell cycle arrest, reminiscent of reserve cells, and are capable of being induced to proliferate and differentiate again. Indeed, cell cycle arrest is one of the cell fates known to be induced by p53, albeit generally requiring only relatively small increases in p53 expression.

Despite its likely role in cell fate decision-making in adult stem and progenitor cells, the dynamical behavior of p53 has been difficult to assess in these cell types, as using live cell microscopy over days is technically challenging with intravital methods. Instead relying on genetic manipulation of p53 in mouse models, studies have shown that p53 promotes quiescence in hematopoietic stem cells (Liu et al., 2009), potentially consistent with our results, but in studies in other adult stem cells, including mammary gland, mesenchymal, and neural stem cells, p53 deficiency can either enhance or oppose pluripotency and self-renewal (Solozobova and Blattner, 2011). Our data corroborate a previous study in muscle satellite cells (Flamini et al., 2018), but to our knowledge this is the first report monitoring p53 dynamics in real time in the progenitor progeny of any adult stem cell type.

For human embryonic stem cells, p53 accumulation due to DNA damaging conditions was shown to correlate with stem cell death (Liu et al., 2013). Evolutionarily, this would likely be beneficial for the maintenance of genome integrity and reduction of detrimental mutations in the developing organisms. However, to trigger cell death, the DNA damage-induced p53 rise in hESCs required the hESC-specific context that pro-apoptotic mitochondrial Bcl-2 family members were highly primed (Liu et al., 2013). Here, in adult myoblasts, we conversely find that elevated p53 conditions associate with the attenuation of differentiation, presumably to return to a quiescent, more stem-like state. Future studies exploring the larger network of cell fate pathways that act in parallel to p53 or downstream of p53 will help in determining similarities and differences in cell fate decisions regulated in embryonic versus adult stem cells. For instance, the MEK/ERK pathway was found in bovine myoblasts to be active during differentiation through RNAseq approaches (Melzener et al., 2024), and interplay between p53 and ERK dynamics has been shown (Hanson and Batchelor, 2022). Further investigation using live cell time-lapse imaging has the potential to yield more insight into pathways interacting with p53 to modulate cell fate decisions during myoblast differentiation.

## Materials and methods

### Cell line construction

Immortalized human skeletal muscle myoblasts (M007) were generously provided by Dr. Darko Bosnakovski and Dr. Michael Kyba (Bosnakovski et al., 2023). Lentiviral transduction was conducted in M007 cells to express a previously generated fluorescently tagged p53 reporter (p53-mVenus) (Batchelor et al., 2008) and G418 resistance. Cells were incubated with concentrated lentivirus overnight (16 h), and 48 h after virus removal and media replacement, media was supplemented with 200 μM G418 to select for cells with transgene expression. Cells were further selected by fluorescence-activated cell sorting (FACS) on a FACSAriaII (BD Biosciences), and the sorted cells with higher yellow fluorescence signal were thereafter separately cultured as the M007 p53-Venus line.

### Cell culture

M007 cells were cultured in F-10 Ham’s medium supplemented with 20% fetal bovine serum (FBS), 1% glutamax, 1% penicillin/streptomycin (P/S), 100 µM 2-mercaptoethanol, 0.0727 µM basic fibroblast growth factor (bFGF), and 0.1 µM dexamethasone (therefore referred to as “M007 media”). For maintenance, both parental M007 cells and M007 p53-Venus cells were passaged at a 1:10 to 1:20 ratio upon reaching approximately 90% confluence. Cultures were not passaged more than 20 times to maintain line stability.

### Time-lapse fluorescence microscopy

Two days prior to microscopy, parental M007 and M007-p53Venus cells were seeded into 12-well glass-bottom plates (MatTek P12G-1.5–14-F) coated with 0.1% gelatin at a density of 300,000 cells per well and allowed to adhere and grow in M007 media. On the day of the microscopy experiment, media was changed to Dulbecco’s Modified Eagle Medium (DMEM) lacking phenol red (Gibco, 11,054-020) with 2% horse serum (Cytiva, SH30074.03) to induce differentiation, and half of the wells were also treated with 15 µM Nutlin-3 (Sigma-Aldrich N6287), a concentration of Nutlin-3 previously found to sustain p53 levels without inducing cell death (Purvis et al., 2012; Harton et al., 2019). Live-cell time-lapse microscopy was conducted using a Nikon TiE microscope equipped with a custom environmental chamber to maintain cells at 37 °C and 5% CO_2_. Images were captured using a ×20 plan apo objective (0.75 NA). Both brightfield and yellow fluorescence images (488–512 nm for the excitation light, 520 nm for the dichroic beam splitter, and 532–554 nm for the emission light; Chroma) were captured every 5 min for 72 h to monitor cell morphology and p53-associated fluorescence dynamics. Multiple positions were imaged within each well to ensure representative sampling across the area.

### Image analysis and clustering

Time-lapse microscopy images were analyzed using the p53Cinema pipeline for image segmentation, cell tracking, and fluorescence quantification (Reyes et al., 2018). Further analysis was performed using standard functions in Matlab. Background fluorescence values were subtracted based on regions devoid of cells in each frame of the microscopy movies for each time point and microscope position. Fluorescent traces for each cell were smoothed using a Gaussian filter. Clustering was performed by first filtering time-lapse traces failing to surpass a 50% threshold of missing data for individual time points across all time points imaged. Of the remaining traces, missing data was imputed from valid time-point data from neighboring time points. Three clusters were explicitly calculated for each condition using K-means clustering.

### Immunofluorescence microscopy

Cells were fixed with 4% paraformaldehyde for 10 min at room temperature, followed by permeabilization with 0.5% Triton X-100 for 5 min and blocking in 5% goat serum for 1.5 h. Subsequently, cells were incubated overnight at 4 °C with primary antibodies against MYH1E (1:50; Developmental Studies Hybridoma Bank, MF20) and p53 (1:500; Proteintech, 10442-1-AP), or cyclin B1 (1:400; Cell Signaling Technologies 4138 S) and p53 (1:500, DO-1 Santa Cruz sc-126) in 1% bovine serum albumin (BSA) in phosphate-buffered saline (PBS). The following day, cells were incubated with secondary antibodies Alexa Fluor 594–conjugated and Alexa Fluor 488–conjugated secondary antibodies (Jackson ImmunoResearch) diluted (1:500) in 1% BSA. Lastly, cells were treated with DAPI (1 μg/mL in PBS) for nuclear staining. Between steps, cells were washed with PBS. Cells were imaged on a Keyence BZ300. Image segmentation for individual cells was performed based on DAPI images, cell phenotype was assayed based on mean MF20 staining, and mean p53 levels were acquired per cell following background subtraction of p53 staining using Fiji scripts (ImageJ).

#### Fluorescence activated cell sorting and cell cycle analysis

M007 p53-Venus cells were grown for 72 h in proliferation or differentiation medium. Cells were harvested by trypsinization and then fixed using Cyto-Fast Fix/Permeability Buffer Set (Biolegend 426,803) following the manufacturer’s protocol. After fixation, cells were incubated in 50 μg/mL propidium iodide (Thermo Fisher Scientific P3566) and 100 μg/mL RNaseA (Thermo Fisher Scientific EN0531) for 1 h at 37 C. Cells were analyzed using a FACS Aria II, sorting for single cell and detecting p53-Venus and propidium iodide fluorescence. Data were analyzed using FlowJo software (FlowJo) and GraphPad Prism (GraphPad).

## Data Availability

The raw data supporting the conclusions of this article will be made available by the authors, without undue reservation.
