# Supplementary material for: Dynamic increases in p53 expression suppress human myoblast differentiation
Source: Front Cell Dev Biol. 2026 Oct 1;14:1844660. doi: 10.3389/fcell.2026.1844660 (PMC13669106; doi:10.3389/fcell.2026.1844660)
Supplement: Supplementary file 1 [file DataSheet1.pdf]

Supplementary Figure S1

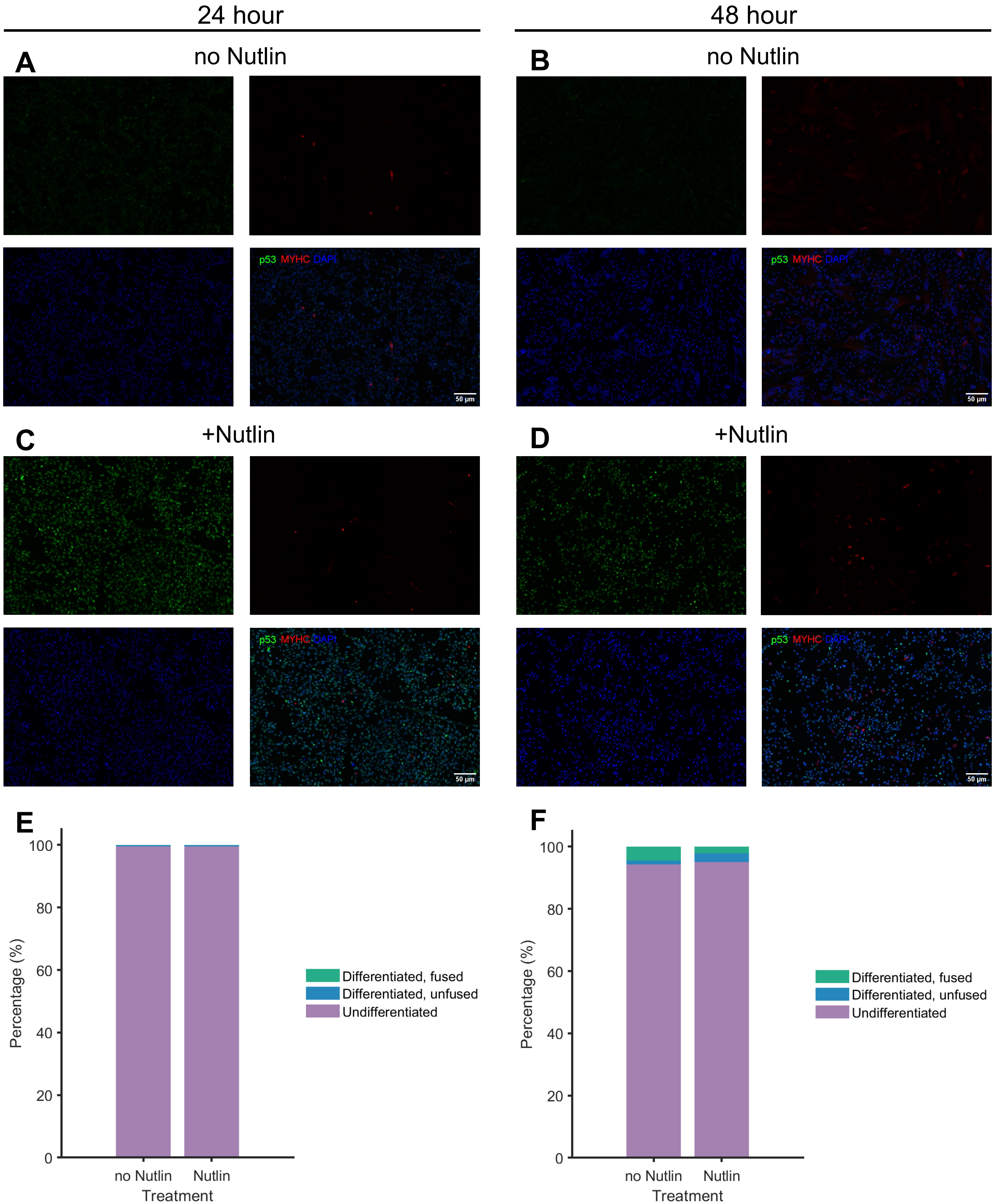

Supplementary Figure S1. (A-B) Immunofluorescence images of M007 p53-Venus cells stained for the muscle marker MYHC (red), p53 (green), and DAPI (blue) for cells treated with only differentiation medium (A, B) or differentiation medium and 15 µM Nutlin-3 (C, D) for 24 h (A, C) or 48 h (B, D). Scale bar = 50 µm. (E-F) Bar plots of the relative cell numbers for cells that were unfused with low MYHC, unfused with high MYHC, or fused with high MYHC for 24 h (E) or 48 h (F) treatment with differentiation medium. Results are for 28,090 cells (24h, -Nutlin), 23,011 cells (24 h, +Nutlin), 16,495 cells (48 h, -Nutlin), and 20,792 cells (48 h, +Nutlin) from biological triplicates.

Supplementary Figure S2

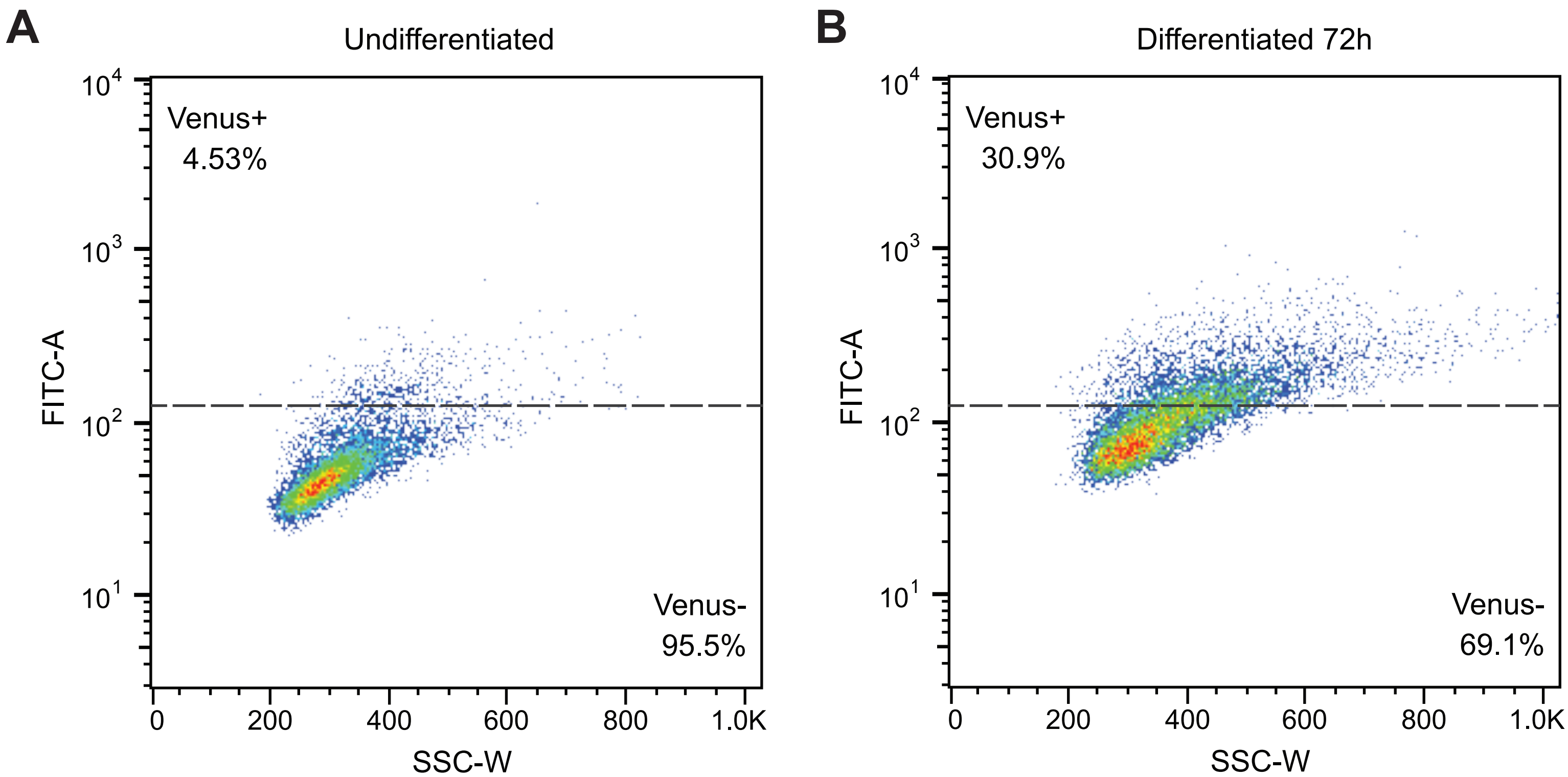

Supplementary Figure S2. Flow cytometry gates for identifying M007 p53-Venus cells expressing elevated p53 expression in (A) normal growth medium or (B) in response to differentiation medium after 72 h.

Supplementary Movie 1. Example time-lapse microscopy brightfield images for M007 p53-Venus cells treated with differentiation medium. Movies are generated from individual frames taken every 5 minutes and show 50 h total of acquisition following treatment with differentiation medium.

Supplementary Movie 2. Example time-lapse microscopy Venus-channel images for M007 p53-Venus cells treated with differentiation medium. Movies are generated from individual frames taken every 5 minutes and show 50 h total of acquisition following treatment with differentiation medium.

Supplementary Movie 3. Example time-lapse microscopy brightfield images for M007 p53-Venus cells treated with differentiation medium supplemented with 15 µM Nutlin-3. Movies are generated from individual frames taken every 5 minutes and show 50 h total of acquisition following treatment with differentiation medium and Nutlin-3.

Supplementary Movie 4. Example time-lapse microscopy Venus-channel images for M007 p53-Venus cells treated with differentiation medium supplemented with 15 µM Nutlin-3. Movies are generated from individual frames taken every 5 minutes and show 50 h total of acquisition following treatment with differentiation medium and Nutlin-3.
